# Chemistry without the Born–Oppenheimer approximation

**DOI:** 10.1098/rsta.2020.0375

**Published:** 2022-05-16

**Authors:** Federica Agostini, Basile F. E. Curchod

**Affiliations:** ^1^ CNRS, Institut de Chimie Physique UMR8000, Université Paris-Saclay, 91405 Orsay, France; ^2^ Centre for Computational Chemistry, School of Chemistry, University of Bristol, Cantock's Close, Bristol BS8 1TS, UK

Nearly a century ago, Born and Oppenheimer published an article on what would rapidly emerge as the cornerstone for our representation of molecules [1]. Their work offered a strategy to decompose the energy of a molecule into several contributions that will soon become a workhorse for spectroscopy: electronic, vibrational and rotational energy (figure 1*a*). Born and Oppenheimer could achieve this decomposition under the assumptions that a stable configuration of the molecule exists and (electronic) degeneracies are absent. Furthermore, the separation of the total molecular energy into the three contributions is only an approximation, and coupling between these terms exists.

**Figure 1.**
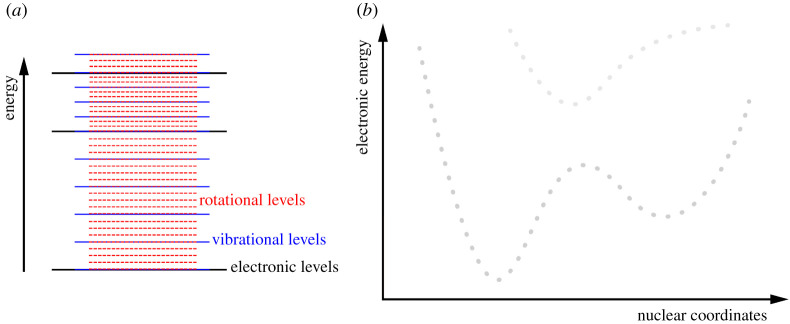
Illustration of the conceptual outcomes triggered by Born and Oppenheimer in their seminal work. (*a*) The total energy of a molecule can be decomposed into the sum of an electronic (black), vibrational (blue) and rotational (red) energy. (*b*) The eigenvalues of the electronic Hamiltonian—the electronic energies—are functions of nuclear coordinates and lead to the concept of *potential energy surfaces*. The Born–Oppenheimer approximation permits the consideration of only one of the potential energy surfaces under certain conditions. (Online version in colour.)

Born and Oppenheimer formulated the following two main conclusions in their work [1]. First, the eigenvalues of the electronic Hamiltonian, i.e. the molecular Hamiltonian without the nuclear kinetic energy operator, are functions of nuclear coordinates and play ‘the role of a potential for the nuclear motion’ (figure 1*b*). Second, the coupling effects among electronic states appear beyond the fourth order in κ, where $\kappa ={\left(m/M\right)}^{1/4}$ plays the role of a small parameter in their use of stationary perturbation theory and m/M is the electron–nuclear mass ratio. While these conclusions may appear technical at first glance, they describe what are perhaps some of the most powerful tools ever developed to date for studying chemical reactivity: the concept of electronic potential energy surfaces and the Born–Oppenheimer approximation. These tools have been used to forge our way of picturing molecules over the last century and are central pillars to the development of theoretical and computational chemistry [2].

In the Born–Oppenheimer approximation, the electrons are forced to remain in a given eigenstate of the electronic Hamiltonian as the nuclear configuration changes. In other words, the nuclei constituting the molecule evolve on a single potential energy surface and are glued by this electronic configuration. This picture resonates with numerous tools used by chemists in their everyday life. Drawing the Lewis structure of a molecule, or speaking about its bonds, relies on the idea that the electrons are attaching the nuclei in a specific way. Similarly, in chemical reactivity chemists connect the energy of stable nuclear configurations—reactants, products—along a reaction coordinate that can contain intermediates and transition states. The Born–Oppenheimer approximation is here once more implied, as one considers only a single potential energy surface, whose stationary points represent the critical steps to characterize chemical reactions. In a dynamical picture, the Born–Oppenheimer approximation introduces a peculiar perspective on the motion of a molecule, suggesting to look at electronic dynamics on a time scale typical of nuclear dynamics [3]. As the electrons are much lighter than the nuclei, i.e. κ→0, the dynamics of the molecule can be decomposed into a time-independent electronic problem coupled to a time-dependent nuclear dynamics. Sometimes, the Born–Oppenheimer approximation is incorrectly associated with the clamped nuclei approximation, where the nuclear kinetic energy is completely neglected in the molecular Hamiltonian. Similarly, the Born–Oppenheimer approximation is sometimes used as a synonym for a classical-nuclei approximation, where the nuclei are treated as classical point particles. However, molecular energies do, even within the Born–Oppenheimer approximation, include a contribution from the nuclear kinetic operator. The quantum-mechanical behaviour of the nuclei is also often crucial to correctly capture their dynamics along a single potential energy surface, for example, to describe tunnelling processes.

The brief discussion above spotlights the role of the cornerstone that the Born–Oppenheimer approximation plays not only in fields like classical and *ab initio* molecular dynamics or quantum chemistry, but more generally in the usual representation that a chemist uses to depict a molecule. Despite the success of the Born–Oppenheimer approximation and its crucial role in chemistry, its domain of validity remains finite and the past decades have seen a growing interest in cases, experimental and theoretical, where this approximation runs out of steam. This theme issue on ‘Chemistry without the Born–Oppenheimer approximation’ endeavours to highlight different typical situations where one needs to reconsider the use of the Born–Oppenheimer approximation and devise new strategies to overcome its limitations. Light-induced processes in photochemistry and photophysics are prime examples of such situations.

## 1. About this theme issue

The opinion pieces opening this theme issue illustrate the importance of chemistry without, and beyond, the Born–Oppenheimer approximation. Ashfold & Kim [4] offer an experimental perspective on the breakdown of the Born–Oppenheimer approximation and present examples highlighting the response of small isolated and solvated molecules to ultraviolet radiation. They focus more specifically on understanding the photostability and photoreactivity of these molecular systems by a concerted experimental and theoretical effort. Hammes–Schiffer [5] proposes an overview on processes involving proton-coupled electron transfer and how they can be addressed by means of non-Born–Oppenheimer dynamics. The study of proton-coupled electron transfer shows how the concepts introduced in the 1927 article by Born and Oppenheimer can be extended to account for the description of electron–proton vibronic states.

The simulation of photochemical processes is a recurring topic in this theme issue, as they constitute a typical example of a breakdown of the Born–Oppenheimer approximation. Light-matter interactions and the ensuing ultrafast, out-of-equilibrium nuclear dynamics are responsible for inducing couplings and transitions among electronic states. The concepts emerging from the article by Born and Oppenheimer can however be used to address these ‘beyond Born–Oppenheimer’, or non-adiabatic, processes and one can introduce multiple, coupled electronic eigenstates and eigenenergies as functions of nuclear positions. Studies on photochemistry of isolated and solvated molecules are often carried out with molecular-dynamics techniques for computational efficiency, so that nuclear dynamics is treated in a trajectory-based fashion coupled to quantum-mechanical electrons described at an *ab initio*, semiempirical or force-field (models and machine-learning) level. Barbatti *et al*. [6] discuss the challenges associated with excited-state dynamics in the long timescales, focusing in particular on the stability of numerical algorithms and the use of machine-learning strategies to move beyond the femto- and picosecond regime in non-adiabatic dynamics. González *et al*. [7] discuss the issue of choosing adequate initial conditions to start non-adiabatic molecular dynamics simulations in combination with a quantum-mechanics/molecular-mechanics set-up to treat solvent effects.

Conical intersections are undoubtedly the most iconic concept belonging to a representation of molecules beyond the Born–Oppenheimer approximation. Born–Oppenheimer potential energy surfaces can, in certain regions of the configuration space, become degenerate. Only a subset of coordinates can lift the degeneracy of the potential energy surfaces, which form a typical double cone that can act as a ‘funnel’—opening efficient pathways between electronic states that a molecule can follow to relax non-radiatively after a photoexcitation. Conical intersections are connected to a singular behaviour of the electronic eigenfunctions in nuclear configuration space, and, as a result, to geometric-phase effects that need to be accounted for within and beyond the Born–Oppenheimer approximation—as long as a Born–Oppenheimer formalism is employed. The contribution by Maskri & Joubert-Doriol [8] discusses geometric-phase effects within a molecular-dynamics approach where the quantum-mechanical nature of the nuclei is pictured by using Gaussian basis functions that evolve according to classical equations of motion.

Describing the dynamics of molecules beyond the Born–Oppenheimer approximation reveals the quantum nature of nuclei. For example, the description of excited-state dynamics through a conical intersection by a trajectory-based approach requires the propagation of an important number of trajectories to reproduce the possible branching of the nuclear wave functions in different electronic states. A great effort has been devoted to developing methods for non-adiabatic molecular dynamics that fully account for nuclear quantum effects, as well as benchmark strategies to assess the validity of approximated models in comparison to numerically exact quantum dynamics. Worth *et al*. [9] propose a comparison between different methods for excited-state dynamics using quantum and classical trajectories, employing the same code and same representation for the electronic states. The contribution by Lauvergnat *et al*. [10] presents an analysis of a complementary issue related to the evaluation of kinetic energy operators and metric tensors for quantum dynamics simulations employing curvilinear coordinates, i.e. chemically intuitive yet non-Cartesian coordinates. Richardson *et al*. [11] discuss in their work the importance of quantum-mechanical effects on the calculation of rate constants in tunnelling regime and beyond the Fermi Golden rule, focusing on the semiclassical instanton theory and its extension beyond the Born–Oppenheimer approximation.

Situations exist where the Born–Oppenheimer approximation provides a very accurate description of the coupled electronic and nuclear dynamics in molecules, but the calculation of observables requires us to account for many electronic states together with their couplings. A striking example is the electronic current (or flux) density, which is always equal to zero within the Born–Oppenheimer approximation. As a consequence, experimental observables depending on this quantity—like the response of a molecule to right and left circularly polarized infrared light, known as vibrational circular dichroism—are incorrectly predicted within the Born–Oppenheimer approximation. These limitations of the Born–Oppenheimer approximation are discussed by Schaupp & Engel [12].

The previous paragraphs made it clear that our representation of chemistry and photochemical processes is highly influenced by the Born–Oppenheimer approximation and its subsequent extensions. Nevertheless, alternative theoretical representations of the stationary and time-dependent molecular Schrödinger equation have been formulated without having recourse to a Born–Oppenheimer picture. In a non-Born–Oppenheimer [13] or pre-Born–Oppenheimer [14] formulation, electrons and nuclei are treated equivalently in molecules to reach high accuracy in predicting molecular energies. The exact factorization is another example of an alternative representation of the molecular wave function. While the molecular wave function of the exact factorization appears to be similar to that of the Born–Oppenheimer approximation, that is, a single product of a nuclear and electronic wave function, the electronic wave function in the exact factorization is time-dependent, meaning that this representation is formally exact and hence capable of describing excited-state processes. The formalism of the exact factorization is discussed by Gross *et al*. [15] focusing on the analysis of the expression of the nuclear kinetic energy in a two-component system.

We hope that the contributions to this theme issue will not only offer a brief, even if far from exhaustive, account of recent developments in chemistry without the Born–Oppenheimer approximation, but also stimulate the curiosity of the reader in this highly active field at the interface between chemistry, physics, mathematics and computer science.

## Data Availability

This article has no additional data.
